# Electrocatalytic oxidation of ethanol at Pd, Pt, Pd/Pt and Pt/Pd nano particles supported on poly 1,8-diaminonaphthalene film in alkaline medium

**DOI:** 10.1039/c7ra13694c

**Published:** 2018-04-24

**Authors:** K. M. Hassan, A. A. Hathoot, R. Maher, M. Abdel Azzem

**Affiliations:** Electrochemistry Research Laboratory, Physics and Mathematics Engineering Department, Faculty of Electronic Engineering, Menoufia University Egypt drkhalidhassan73@gmail.com +201001303945; Electrochemistry Laboratory, Chemistry Department, Faculty of Science, Menoufia University Egypt

## Abstract

An ethanol oxidation reaction (EOR) in alkaline medium was carried out at palladium (Pd) or platinum (Pt) nanoparticles/poly 1,8-diaminonaphthalene (p1,8-DAN) composite catalyst electrodes. Pd and Pt were incorporated onto a p1,8-DAN/GC electrode by a cyclic voltammetry (CV) strategy. The obtained Pd/p1,8-DAN/GC, Pt/p1,8-DAN/GC, Pt/Pd/p1,8-DAN/GC and Pd/Pt/p1,8-DAN/GC modified electrodes were characterized by scanning electron microscopy (SEM), energy dispersive X-ray spectroscopy (EDX) and cyclic voltammetry (CV) techniques. Electrode surface areas (ESAs) of the obtained catalysts were calculated by carbon monoxide (CO) adsorption using differential electrochemical mass spectroscopy (DEMS). The electrocatalytic oxidation of ethanol (EtOH) at the catalyst electrodes was considered in 0.5 M NaOH solutions by CV and chronoamperometric techniques. The catalyst electrodes significantly enhanced the catalytic efficiency for EOR compared to a bare glassy carbon (GC) electrode. Bimetallic catalyst electrodes demonstrate improved catalytic activity, superior durability and higher tolerance to (CO) poison generated in the development of EOR compared with Pd/p1,8-DAN and Pt/p1,8-DAN catalysts, giving priority to Pt/Pd/p1,8-DAN/GC electrodes. Viability parameters, such as NaOH and EtOH concentrations, scan rate and upper potential limits, were examined and analyzed. This study suggests that the prepared catalysts have pronounced potential applications in direct EOR in fuel cells.

## Abstract 2

An ethanol oxidation reaction (EOR) in alkaline medium was carried out at palladium (Pd) or platinum (Pt) nanoparticles/poly 1,8-diaminonaphthalene (p1,8-DAN) composite catalyst electrodes.

## Graphical abstract

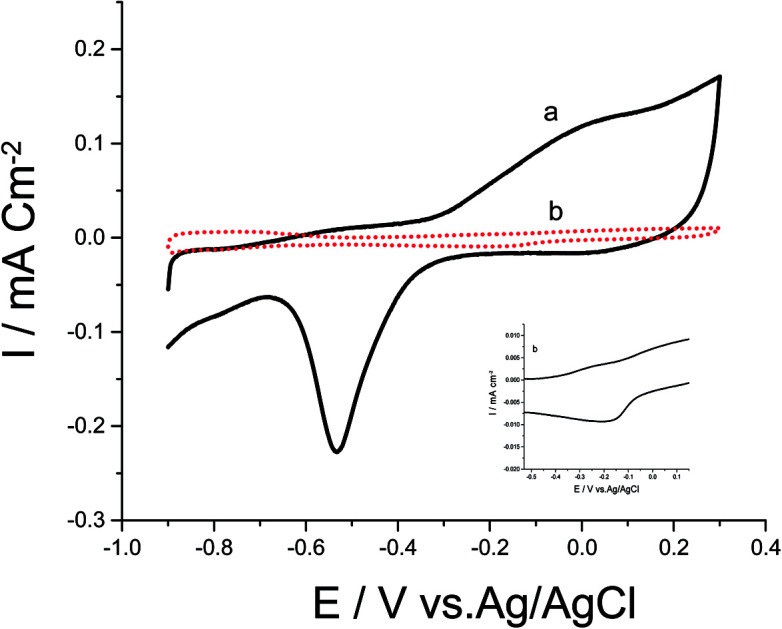

## 1. Introduction

In recent decades, direct oxidation fuel cells have pulled in extraordinary consideration since they offer a productive and green innovation for energy conversion. Nevertheless, the utilization of hydrogen is restricted by issues of fabrication, purification, storage and distribution. Alkaline direct oxidation fuel cells engaging on several liquid fuels promise to be an environmentally friendly energy technology, mainly because of the enhanced performance as a result of fast electrochemical kinetics on both the anode and cathode.^1–7^ Among several liquid fuels, ethanol (EtOH) has been documented to be the most appropriate fuel as it is a sustainable and carbon-neutral transport fuel.^8^ Insignificant poisoning effects in alkaline solutions were also detected.^6^ In electrochemical oxidation, the electrode material is a fundamental factor, and a highly effective electrocatalyst is required. Conducting polymer matrixes with excellent stability, a porous nature and high active surface area (like poly 1,8-diaminonaphthalene) are attractive and favorable supports for incorporation of the catalyst nanoparticles (NPs).^9–11^ The high available surface area, the synergistic impact between NPs and the conducting polymer and the improved tolerance against poisoning by adsorbed carbon monoxide (CO_ads_), which is formed during the EtOH electrooxidation, enhance the effectiveness of the electrocatalyst and reduce the surface poisoning. The supported structure prevents agglomeration and affords a high degree of distribution of MNPs during the electrodeposition for liquid fuel oxidation. It could be considered as a redox mediator between the electrode and the electroactive reactant by electron–proton exchange. Composites of conducting polymers with NPs offer a rapid electron transfer pathway through the polymer layer during the electrochemical process. Therefore, metal electrodeposition on the conducting polymers can provide a low-cost and appropriate approach for electrode modification.^12–14^ Pt has been broadly used as the anode catalyst for liquid fuel oxidation due to its excellent electrocatalytic capability.^15–17^

Disregarding the critical changes made up to now in the performance of direct oxidation fuel cells, various technical obstacles to their marketable application have continued, including the low performance of the anodes for the oxidation process and the high cost of noble metal platinum-based (Pt-based) catalysts.^18–20^ Diverse methodologies have been utilized to help the commercial application of direct oxidation fuel cells: for example, the incorporation of another metal into Pt, controlling the catalyst morphology, and choosing appropriate support materials.^11,21,22^

The incorporation of a second metallic element into Pt has been recognized to help the development of liquid fuel oxidation kinetics considerably.^9,11,12,23^ Referring to the dual-function and fundamental mechanism, a second metal can provide oxygen containing species at lower potentials and reduce the bond strength of Pt–CO_ads_, and then support the oxidation removal of CO to CO_2_.^21^ Palladium (Pd) has been used as an alternative material to Pt, mainly in fuel cells, due to its reasonably abundant source and proper electrocatalytic activity.^6,21,24^ There are few reports on utilizing conducting polymers as dispersion media for Pt and Pd NPs. In this paper, we report a simple strategy for the preparation of a poly 1,8-diaminonaphthalene/glassy carbon modified electrode as an effective support to anchor single and bimetallic Pd and Pt NPs.^25^ This material offers a high potential for an electrocatalyst support for an EtOH oxidation reaction (EOR).

## 2. Experimental

### 2.1. Materials

1,8-DAN of analytical grade obtained from Aldrich was conserved in the dark and stored in a refrigerator before use. Sulfuric acid (H_2_SO_4_ 98%) (Merck), acetonitrile (ACN) (99.9%), HPLC (LAB-SCAN) and EtOH (ADWIC) (98%) were used without additional purification. Sodium hydroxide (NaOH), palladium chloride (PdCl_2_) and chloroplatinic acid hexahydrate (H_2_PtCl_6_·6H_2_O) were analytical grade chemicals. Diamond paste (Presi) 2.0 μm and freshly distilled water were used continuously.

### 2.2. Instruments

SEM images were recorded using QUANTA FEG 250 equipped with an energy dispersive ray spectrometer (EDS). Electrochemical measurements were recorded using a potentiostat Model BASi EPSILON. All electrochemical experiments were achieved using an electrochemical cell, with a conventional three-electrode system. A GC electrode (3.0 mm diameter) was used as the working electrode and Pt wire as an auxiliary one. All reported potentials were recorded with respect to an Ag/AgCl reference electrode. Electrochemical surface area (ESA) measurements were carried out using an EG&G potentiostat (model 273A) in combination with LabVIEW software (National Instruments GmbH, Munich, Germany) for recording cyclic voltammograms. Carbon dioxide (CO_2_) was detected by differential electrochemical mass spectroscopy (DEMS) using a quadrupole mass spectrometer (Balzer QMG-422) with dual thin layer flow through a cell in which a hydrophobic Teflon membrane forms the interface between the electrolyte and the vacuum.

### 2.3. Experimental procedures

#### 2.3.1. Electrochemical preparation of p1,8-DAN modified electrode

Formation of p1,8-DAN film was carried out in a mixed solvent of 4.5 M H_2_SO_4_ in ACN in the presence of 1.0 mM DAN monomer at a GC electrode using the CV technique. The electrode potential was swept at a rate of 0.1 V s^−1^ between 0.2 and 1.2 V for 20 cycles.^26^

#### 2.3.2. Metal nanoparticles (MNPs) electro-deposition

A monometallic Pd or Pd catalyst prepared by immersing a p1,8-DAN modified electrode in an aqueous solution of 0.1 M H_2_SO_4_ containing 2.5 mM PdCl_2_ or 0.1 M HClO_4_ containing 2.5 mM H_2_PtCl_2_ produced Pd/p1,8-DAN/GC or Pt/p1,8-DAN/GC catalyst electrodes, respectively. The CV technique was used for MNPs electro-deposition by cycling the potential between −0.35 and +0.65 V at a scan rate of 0.05 V s^−1^ for 25 cycles.^27^ Bimetallic Pd/Pt or Pt/Pd similarly deposited onto the p1,8-DAN/GC modified electrode using the same method finally produced Pd/Pt/p1,8-DAN/GC or Pt/Pd/p1,8-DAN/GC catalyst electrodes.

#### 2.3.3. ESA measurements

Electrode surface areas measurements (ESAs) of Pd/p1,8-DAN/GC, Pt/p1,8-DAN/GC, Pd/Pt/p1,8-DAN/GC and Pt/Pd/p1,8-DAN/GC catalysts were achieved by the DEMS technique where CO was adsorbed at a constant electrode potential of 0.06 V by flowing a CO saturated 0.1 mM H_2_SO_4_ supporting electrolyte. After formation of the CO monolayer, the solution was replaced by a pure 0.5 M supporting electrolyte under potential control (*E* = 0.06 V) in order to maintain a solution free of CO. The faradic and ionic currents were recorded during the positive potential sweep at a scan rate of 0.01 V s^−1^ and a flow rate of 5 μL s^−1^.

## 3. Results and discussion

### 3.1. Electrochemical performances and surface morphology of the catalysts

**Fig. 1:**
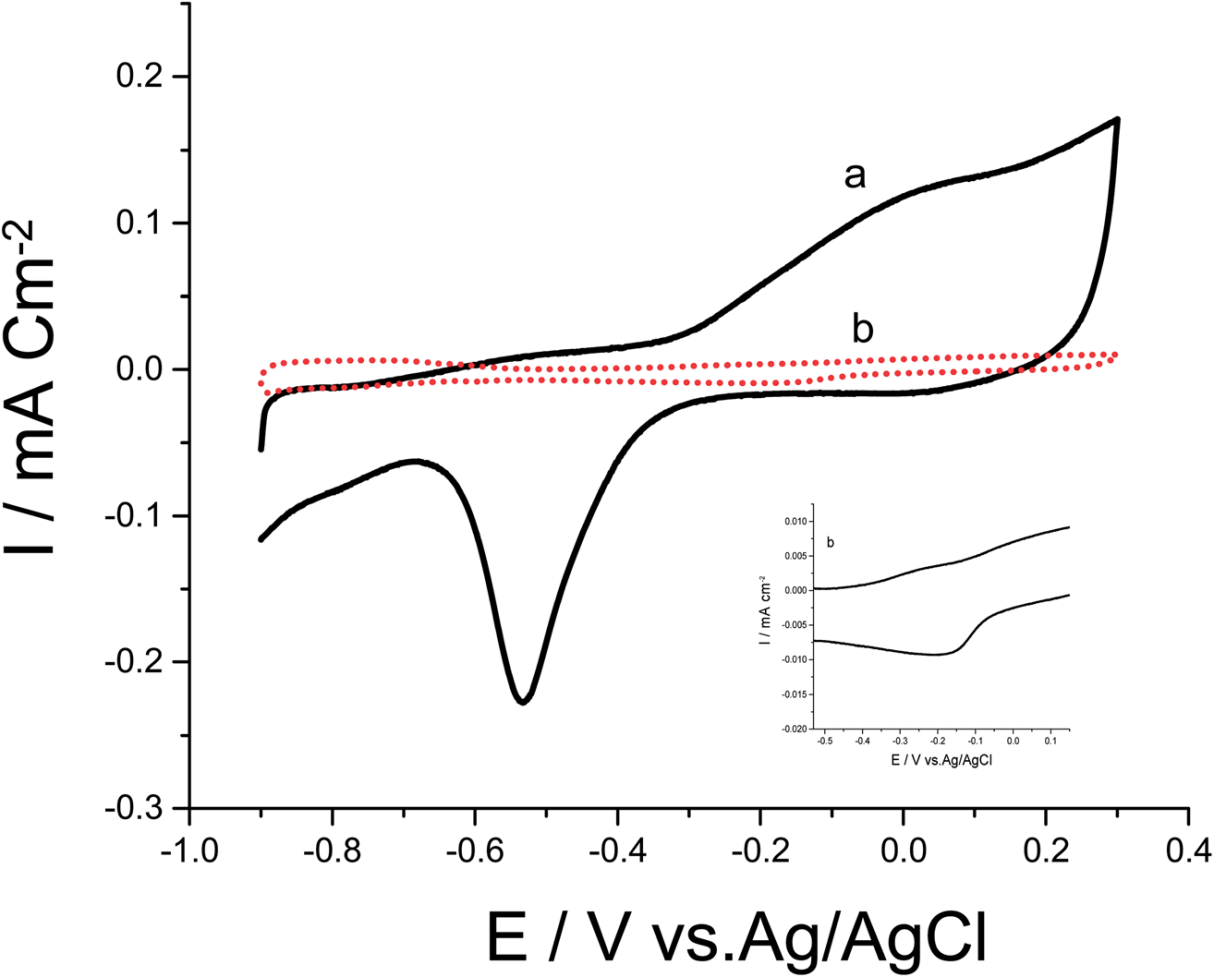
CVs of (a) Pd/p1,8-DAN/GC and (b) Pt/p1,8-DAN/GC modified electrodes in 0.5 M NaOH at a scan rate of 0.05 V s^−1^.

Cyclic voltammograms (CVs) of both Pd/p1,8-DAN/GC and Pt/p1,8-DAN/GC catalyst electrodes in 0.5 M NaOH in the potential range of −0.9 V to 0.3 V were recorded at scan rate of 0.05 V s^−1^, as shown in Fig. 1. In the forward scan, oxidation of Pd or Pt forming an oxide layer was observed in a potential range from 0.0 V to 0.3 V. In the backward sweep, the oxide stripping peaks for Pd/p1,8-DAN/GC and Pt/p1,8-DAN/GC electrodes were revealed at −0.53 V and −0.15 V, respectively.^24,27,28^

**Fig. 2:**
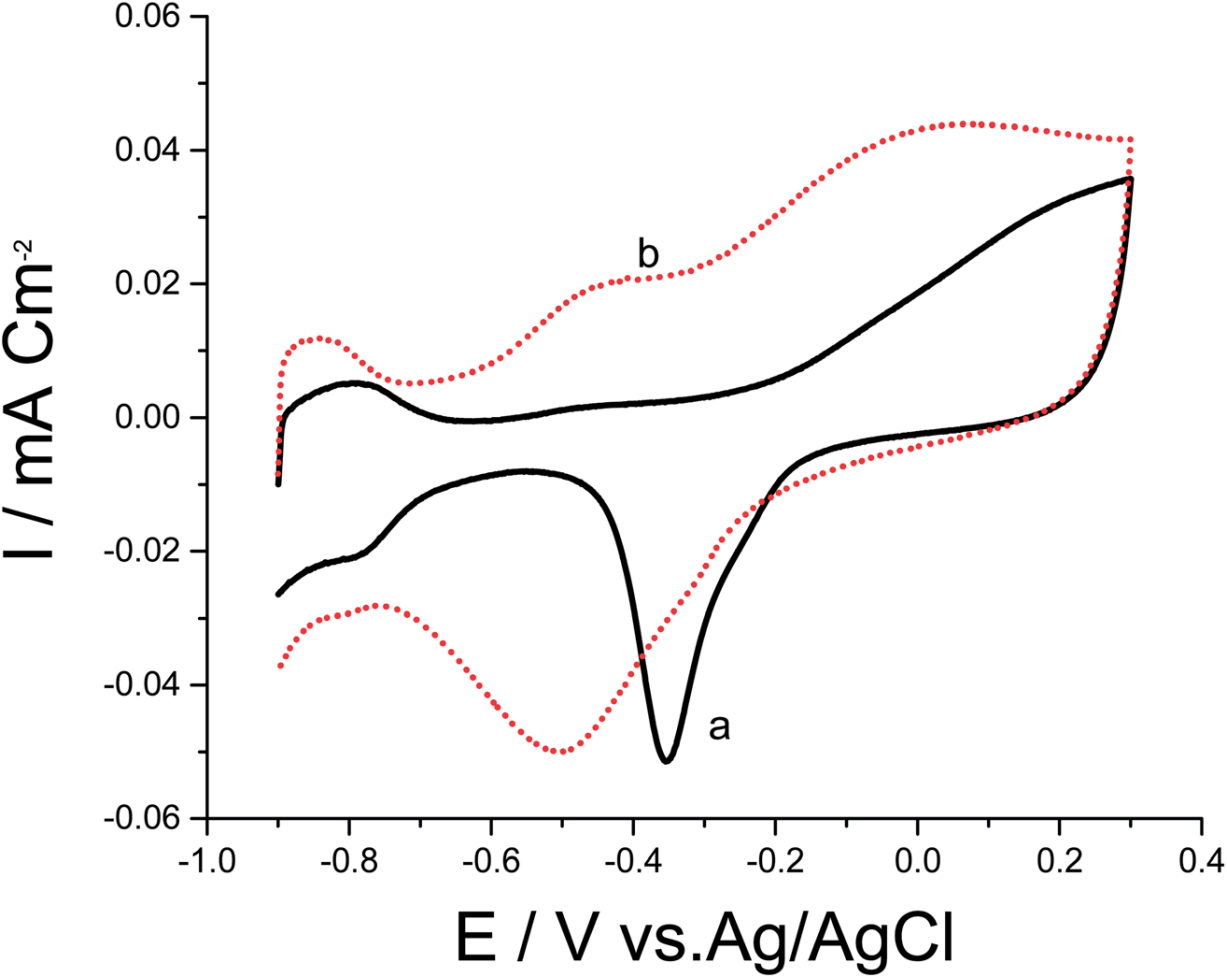
CVs of (a) Pd/Pt/p1,8-DAN/GC and (b) Pt/Pd/p1,8-DAN/GC modified electrodes in 0.5 M NaOH at a scan rate of 0.05 V s^−1^.

To date, a lot of bimetallic materials with synergistically improved activities have been considered based on Pt in combination with its neighboring transition metals. Among these materials, a Pt/Pd catalyst is more stable than other bimetallic catalysts at high potentials.^29,30^ The electrochemical behaviors of bimetallic Pd/Pt/p1,8-DAN/GC and Pt/Pd/p1,8-DAN/GC catalysts were investigated in 0.5 M NaOH in the potential range of −0.9 V to 0.3 V at a scan rate of 0.05 V s^−1^, as exhibited in Fig. 2. Both catalysts demonstrated characteristic anodic peaks for metal oxide formation in the forward sweep, whereas the cathodic peaks at −0.35 V and −0.5 V were due to the oxide stripping of the Pd/Pt/p1,8-DAN/GC and Pt/Pd/p1,8-DAN/GC electrodes, respectively.

ESAs of the prepared catalysts were computed by recording faradic and ionic currents for the oxidation of CO_ads_ to CO_2_ by CV and DEMS procedures (figure not shown). Anodic oxidation peaks appearing at 0.9 V for Pd/p1,8-DAN/GC, and at 0.7 V for the other three catalysts were ascribed to the combined impact of oxidation of CO_ads_ and the partial surface oxidation of Pt or Pd to metal oxide (PtO or PdO). Cathodic reduction peaks shown in the reverse scan were credited to metal oxide reductions. Charges associated with the metal oxide reductions were subtracted from the anodic charges and the charge corresponding to CO_ads_ oxidation.^31–33^ The ionic signal for *m*/*z* = 44 in 0.5 M H_2_SO_4_ at a scan rate of 0.01 V s^−1^ at a flow rate of 5 μL s^−1^ was utilized for ESA calculations according to the following equation:1
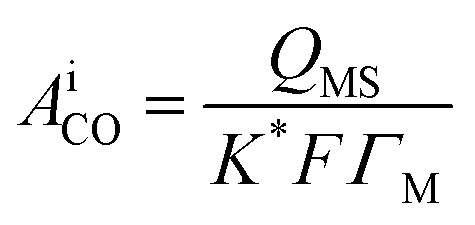
where *F* is the Faraday constant, *Γ*_M_ is the surface concentration of the CO_ads_ monolayer (assuming a *Γ*_M_ of 1.45 nmol cm^−2^ corresponding to 280 μC cm^−2^) and K* is the calibration constant measured by CO stripping on polycrystalline Pt at a scan rate of 10 mV s^−1^.

The acquired ESAs of the catalyst electrodes were 0.2, 2.03, 1.51 and 1.88 cm^2^ for Pd/p1,8-DAN/GC, Pt/p1,8-DAN/GC, Pd/Pt/p1,8-DAN/GC and Pt/Pd/p1,8-DAN/GC modified electrodes, respectively. The results revealed that the Pt/p1,8-DAN/GC modified electrode has the largest ESA while Pd/p1,8-DAN/GC has the lowest. The ESA of the Pd/p1,8-DAN/GC modified electrode was enhanced by the presence of the second metal layer detected in the Pt/Pd/p1,8-DAN/GC modified electrode. These outcomes showed the significant role of the order of electro-deposition of the MNPs. The higher electroactive surface area makes the modified electrode a suitable catalyst.

To compare the activities of different catalysts in terms of profitable efficiency, the current is generally normalized by the mass of loaded metal. Despite the fact that the mass-current density characterizes the economic efficiency of a catalyst, this does not consider the surface area of active metal sites. Electrochemical active surface area (ECSA) is an essential parameter that explains the number of electrochemical active sites with reference to the mass of noble metal^34,35^ as follows:

2 ECSA = *Q*/*sl*

where *Q* is the coulombic charge of the metal oxide reduction peak; *s* is the proportionality constant that correlates charge with area (0.405 mC cm^−2^) and *l* is the electrocatalyst loading (g m^−2^). The metal loading and ECSA of the modified electrodes were computed and are listed in Table 1. Pt/Pd/p1,8-DAN/GC showed more active reaction centers than the other electrocatalysts, an important parameter for their electrocatalytic activity due to its higher ECSA. Generally, the addition of the second metal nano particles to obtain the bimetallic catalysts increased the ECSA values.

**Table 1:** Monometallic and bimetallic catalyst characteristics

ECSA (m^2^ gm^−1^)	Metal loading (gm m^−2^)	Catalyst
7.2	17.13	Pd/p1,8-DAN/GC
9.21	7.21	Pt/p1,8-DAN/GC
25.69	4.97	Pd/Pt/p1,8-DAN/GC
157.58	3.447	Pt/Pd/p1,8-DAN/GC

**Fig. 3:**
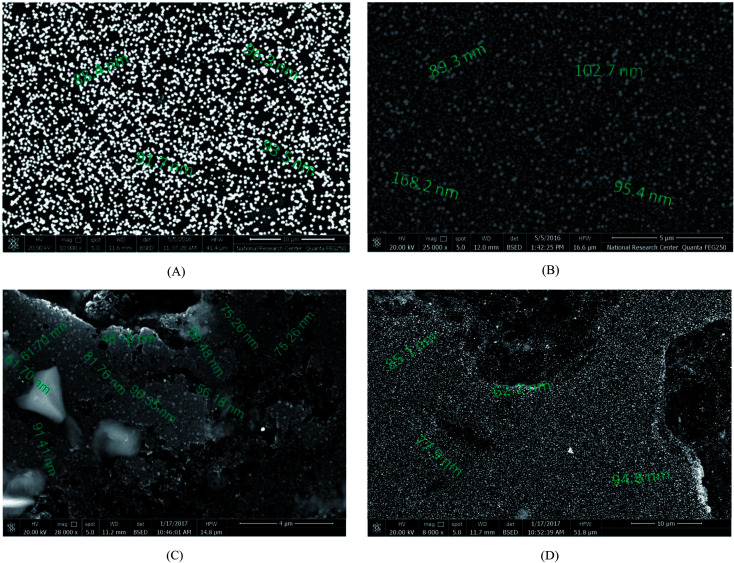
SEM micrographs of freshly prepared catalyst electrodes: (A) Pd/p1,8-DAN/GC, (B) Pt/p1,8-DAN/GC, (C) Pt/Pd/p1,8-DAN/GC and (D) Pd/Pt/p1,8-DAN/GC.

The surface topographies of the obtained catalysts were evaluated using SEM, as shown in Fig. 3A–D, which displays significant differences in the surface structures of the four catalysts.

Electrodeposited Pd (shiny particles) and Pt (light-grey) particle sizes are in the range of 88.4–96.3 nm and 89.3 nm to 168.2 nm, respectively, as shown in Fig. 3A and B. Fig. 3C displays good distributions of Pd and Pt particles, where Pd is in the core and Pt is outside over the whole surface of the p1,8-DAN/GC modified electrode in a size range of 61.7–96.35 nm. When the order of deposition is changed, the aggregated particles are formed in almost the same range, as shown in Fig. 3D.

Furthermore, EDX analysis was conducted (figure not shown) where both Pt and Pd NPs in the monometallic catalysts were dispersed in the p1,8-DAN film with percentages of 5.01% and 0.9%, respectively. Moreover, in the bimetallic layer, EDX results revealed the presence of both dispersed MNPs in the order of 0.07, 0.42% and 0.36, 0.37%, for Pd/Pt/p1,8-DAN/GC and Pt/Pd/p1,8-DAN/GC catalyst electrodes, respectively.

### 3.2. Electrocatalytic activities of the catalysts toward EOR

**Fig. 4:**
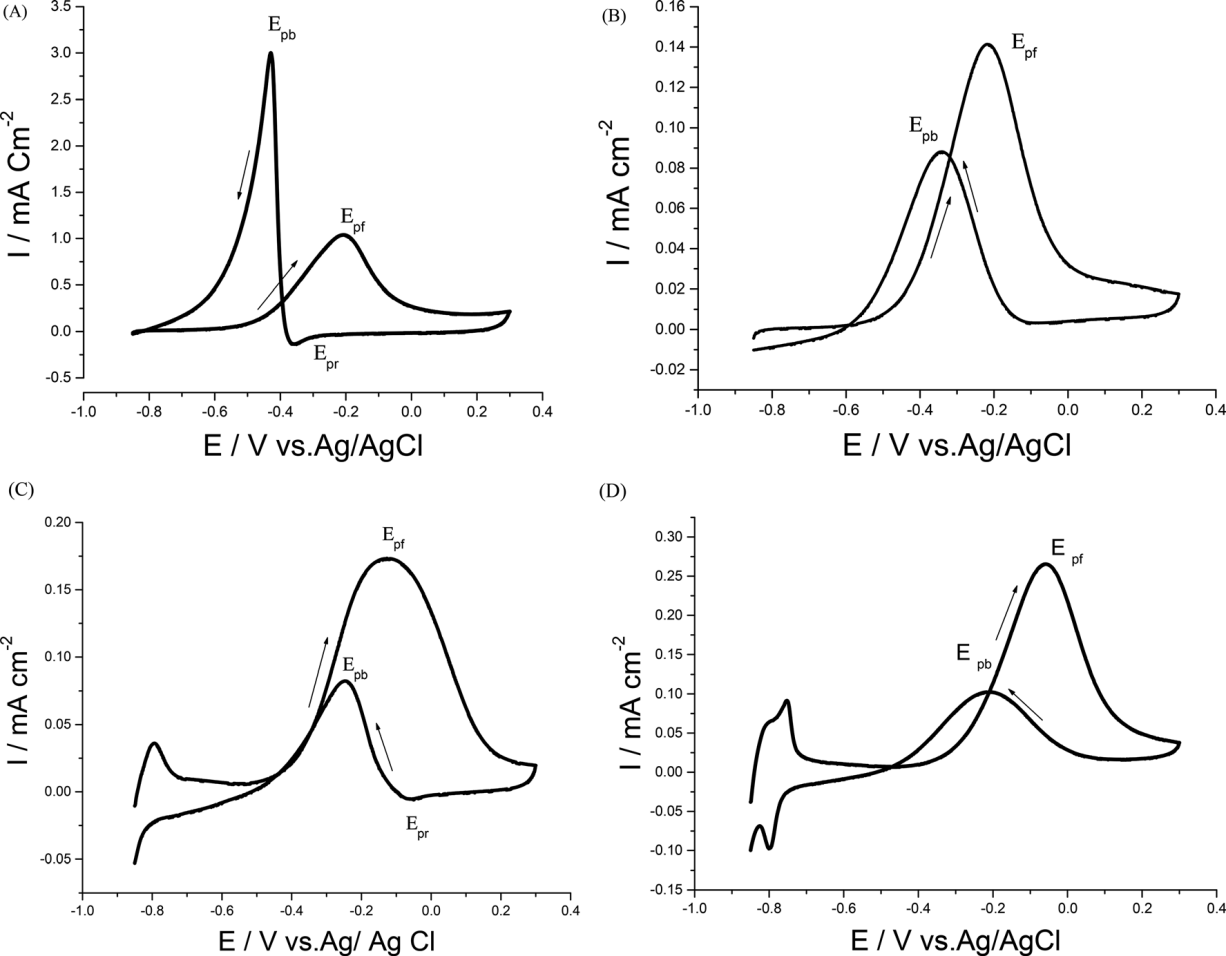
CVs of 0.7 M EtOH in 0.5 M NaOH at (A) Pd/p1,8-DAN/GC, (B) Pt/p1,8-DAN/GC, (C) Pd/Pt/p1,8-DAN/GC and (D) Pt/Pd/p1,8-DAN/GC catalyst electrodes at a scan rate of 0.05 V s^−1^.

Electrocatalytic performances of a bare GC electrode, p1,8-DAN, Pd/p1,8-DAN, Pt/p1,8-DAN, Pd/Pt/p1,8-DAN and Pt/Pd/p1,8-DAN catalyst electrodes toward EOR were assessed by the CV procedure in 0.5 M NaOH containing 0.7 M EtOH at a sweep rate of 0.05 V s^−1^. Typical CV profiles indicated that the GC electrode and p1,8-DAN modified electrode had no electrocatalytic activities. On the other hand, at Pd/p1,8-DAN/GC, Pt/p1,8-DAN/GC, Pd/Pt/p1,8-DAN/GC and Pt/Pd/p1,8-DAN/GC catalyst electrodes, two well-characterized oxidation peaks in the forward and reverse scans (*E*_pf_ and *E*_pb_, respectively)^6^ appeared at −0.20 V and −0.44 V, −0.20 V and −0.35 V, −0.12 V and −0.24 V, and −0.06 V and −0.22 V, respectively (Fig. 4A–D). These oxidation peaks could be ascribed to EtOH electrooxidation.^8,19,36–38^ It is important to mention that the reduction peak (*E*_pr_) of Pd oxide at Pd/p1,8-DAN was observed at a lower potential (*E*_pr_ = −0.35 V)^8^ than that at Pd/Pt/p1,8-DAN (*E*_pr_ = −0.075 V), indicating that single PdNPs oxide is more easily reduced than binary MNPs oxide.^19^ Several significant differences were observed:

(1) Hydrogen adsorption/desorption regions were significantly depressed at Pd/p1,8-DAN and Pt/p1,8-DAN because of surface blocking. This behavior was detected at Pd/Pt/p1,8-DAN and Pt/Pd/p1,8-DAN catalysts in the region between −0.85 and −0.7 V. This could be attributed to the presence of free NPs at bimetallic catalyst surfaces.^19,39–43^

(2) The current density and ECSA were different due to the surface area and the charge of the catalyst electrodes.

(3) *E*_pf_ and *E*_pb_ varied with the metal type of the supported catalyst, giving lower values at Pt/Pd/p1,8-DAN to reach −0.06 and −0.22 V, respectively, as presented in Table 2.

**Table 2:** Electrochemical characteristics of EOR at different catalysts

Catalyst	Current density^a^ (*J*/mA cm^−2^)		Current density^b^ (*j*/mA gm^−1^)		*E*_onset_ (V) *vs.* Ag/AgCl	*E*_pf_ (V) *vs.* Ag/AgCl	*E*_pb_ (V) *vs.* Ag/AgCl	Tolerance
	*J*_pf_	*J*_pb_	*j*_pf_	*j*_pb_				
Pd/p1,8-DAN/GC	1.02	2.93	0.028	0.08	−0.53	−0.20	−0.44	0.35
Pt/p1,8-DAN/GC	0.14	0.08	0.03	0.0171	−0.53	−0.20	−0.35	1.75
Pd/Pt/p1,8-DAN/GC	0.17	0.08	0.01	0.0047	−0.45	−0.22	−0.24	2.13
Pt/Pd/p1,8-DAN/GC	0.26	0.1	0.003	0.00115	−0.39	−0.06	−0.22	2.6

a From DEMS calculation.

b From mass active calculation.

(4) In the EOR mechanism at Pt and Pd catalysts it is considered that the onset potential (*E*_onset_) is related to the breaking of C–H bonds and the consequent removal of intermediates such as CO_ad_ by oxidation with adsorbed OH^−^ (OH_ad_^−^) provided by Pd–OH and/or Pt–OH sites.^27,44^ Also, *E*_onset_ for EOR is an essential factor for determining the electroactivity of the catalyst. At Pd/p1,8-DAN and Pt/p1,8-DAN, *E*_onset_ had similar values of around −0.53 V while it moved in a positive direction for both Pd/Pt/p1,8-DAN and Pt/Pd/p1,8-DAN, to reach −0.45 V and −0.39 V, respectively.

(5) The ratio of the current density value of the forward oxidation peak to that of the backward oxidation peak was utilized to determine the tolerance of the prepared catalysts to accumulated carbonaceous intermediates generated during EOR on the electrode surface.^34^ A higher tolerance ratio demonstrates greater efficiency of EOR during the forward scan and less accumulation of carbonaceous residues on the electrode surface.^27^ Tolerance ratios for the four studied catalysts were computed and are collected in Table 2. It was discovered that the tolerance order was Pt/Pd/p1,8-DAN > Pd/Pt/p1,8-DAN > Pt/p1,8-DAN > Pd/p1,8-DAN. The improved catalytic activity of bimetallic over pure metals is generally ascribed to several effects. It is considered that the alloying component tends to leach out under electrochemical conditions and results in a surface rich in noble metal.^45^ Consequently, it produces an additional active surface compared to monometallic alone. The alloy has also changed the geometric ligand (*e.g.* diminishes the Pt–Pt bond distance) or electronic impact (*e.g.* increase in Pt d-electron vacancies), so one of the components modifies the electronic properties of the other to yield a more active catalytic surface.^46–48^

These results demonstrated that the Pt/Pd/p1,8-DAN catalyst has the highest electrocatalytic activity (the lowest *E*_onset_) and the most elevated tolerance ratio. This could be ascribed to the synergetic impacts of Pt/Pd NPs for the breaking of the C–C bond of EtOH. Additionally, the presence of p1,8-DAN as a highly conducting polymer is an effective support for the catalyst. Therefore, the presence of a support plays an important role in the activity of Pd and Pt NPs towards the EOR, suggesting that Pt/Pd/p1,8-DAN is a good electrocatalyst for EOR in an alkaline medium.^26^

### 3.3. Parameters affecting EOR

In order to achieve better electrocatalytic EOR, different parameters like NaOH and EtOH concentrations, scan rate, and upper potential limits were examined.

#### 3.3.1. Effect of NaOH concentration

**Fig. 5:**
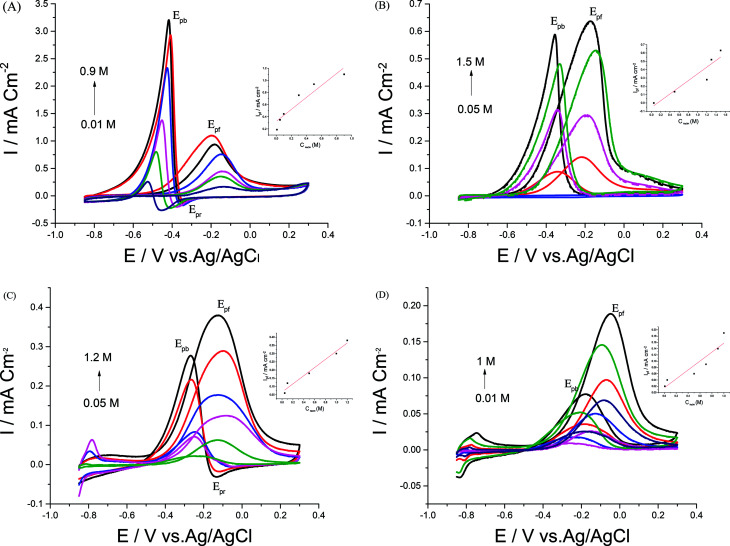
CVs of 0.5 M EtOH in the presence of different NaOH concentrations at (A) Pd/p1,8-DAN/GC, (B) Pt/p1,8-DAN/GC, (C) Pd/Pt/p1,8-DAN/GC and (D) Pt/Pd/p1,8-DAN/GC catalyst electrodes at a scan rate of 0.05 V s^−1^.

Fig. 5A–D indicate distinctive CVs for the four catalysts in the presence of 0.5 M EtOH and different NaOH concentrations. The information obtained demonstrated that the current densities of the forward and backward peaks increased, *E*_pf_ together with *E*_onset_ values shifted to more negative values and the tolerance ratio diminished with increasing NaOH concentration, while *E*_pb_ had a positive shift with a linear dependence and a correlation coefficient of 0.98 (Fig. 5 inset). It was concluded that increasing OH^−^ ion concentration facilitated EOR due the removal of the adsorbed intermediates and the high coverage of catalysts with OH^−^.^27^

#### 3.3.2. Effect of EtOH concentration

**Fig. 6:**
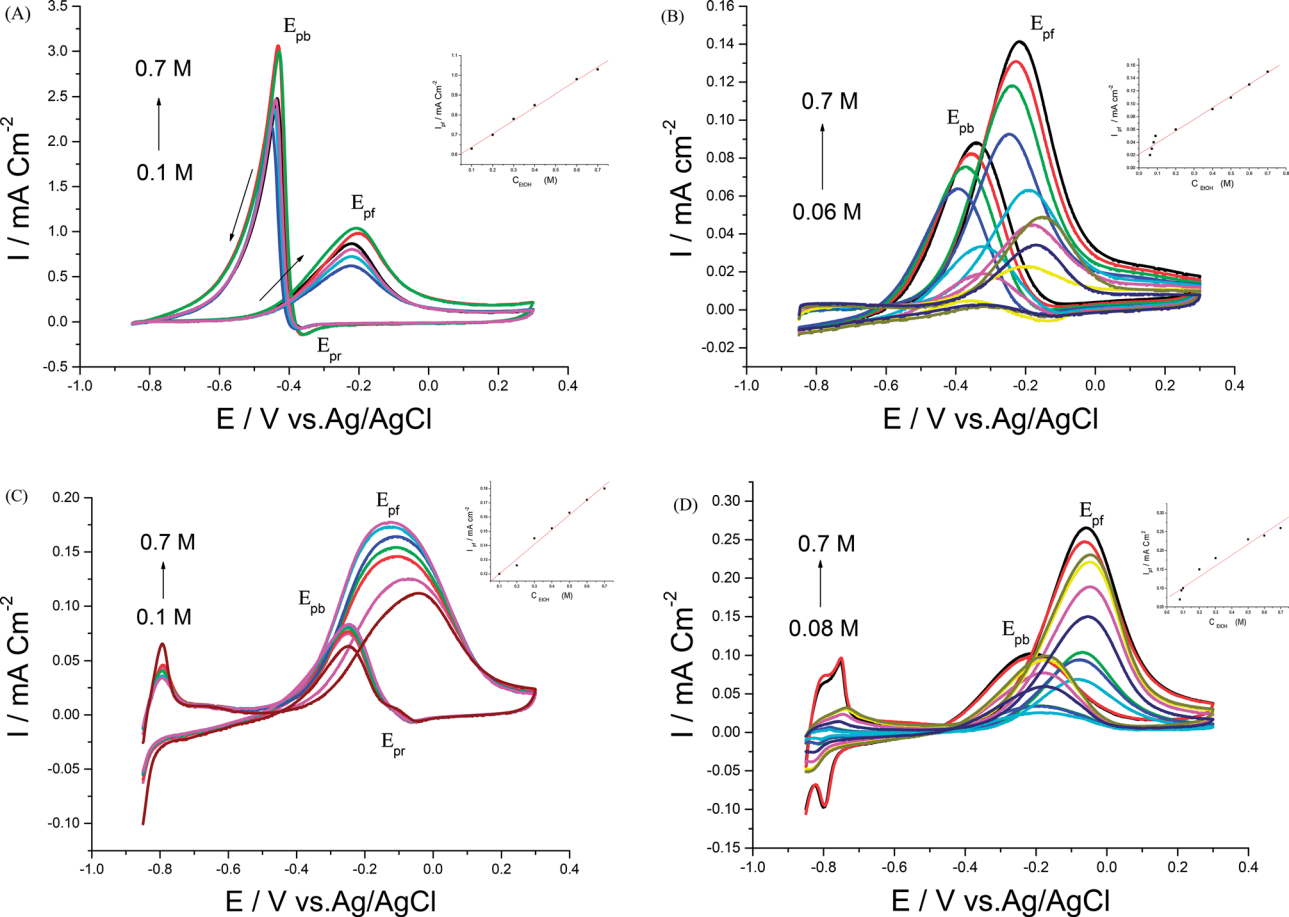
CVs of different EtOH concentrations in 0.5 M NaOH at (A) Pd/p1,8-DAN/GC, (B) Pt/p1,8-DAN/GC, (C) Pd/Pt/p1,8-DAN/GC and (D) Pt/Pd/p1,8-DAN/GC catalyst electrodes at a scan rate of 0.05 V s^−1^.

Different CVs were recorded in 0.5 M NaOH in the presence of various EtOH concentrations, as presented in Fig. 6A–D. As indicated by the experimental data, rising EtOH concentration results in an increase in current density values (Fig. 6 inset) and linear dependence with a correlation coefficient of 0.99. The reaction order for EOR was calculated by plotting the logarithm of EtOH concentration against the logarithm of current density (figure not shown). Reaction orders were 0.47, 0.62, 0.55 and 0.25 at Pd/p1,8-DAN, Pt/p1,8-DAN, Pd/Pt/p1,8-DAN and Pt/Pd/p1,8-DAN, respectively.^27,44,49^ Lower values of the reaction order suggested that the electrooxidation reactions are not limited by reactant concentration (diffusion) and depend mainly on the adsorption steps.^27^

#### 3.3.3. Effect of scan rate

**Fig. 7:**
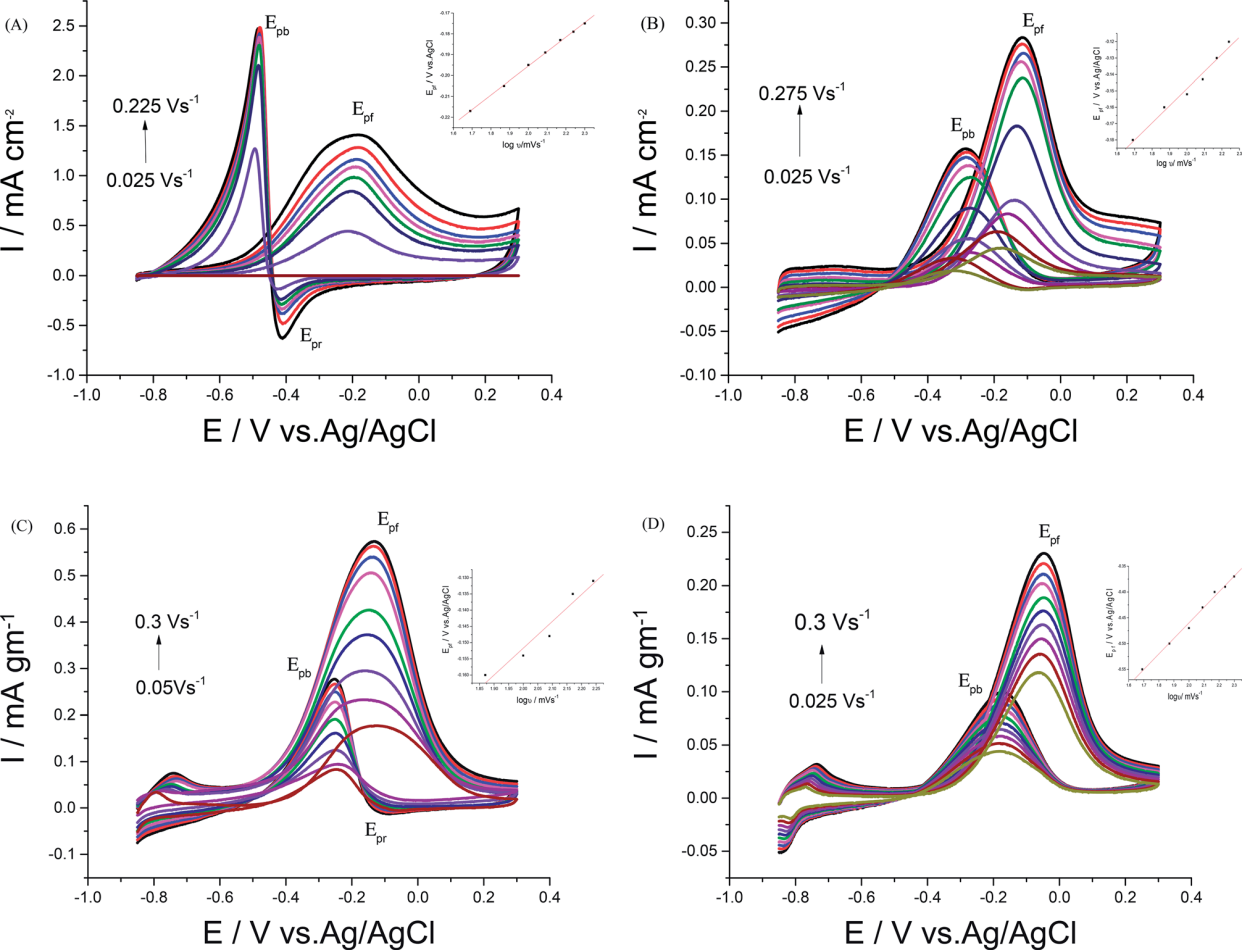
CVs of 0.5 M EtOH in 0.5 M NaOH at (A) Pd/p1,8-DAN, (B) Pt/p1,8-DAN, (C) Pd/Pt/p1,8-DAN/GC and (D) Pt/Pd/p1,8-DAN/GC catalyst electrodes at different scan rates.

The dependence of EOR on transport characteristics at Pd/p1,8-DAN, Pt/p1,8-DAN, Pd/Pt/p1,8-DAN and Pt/Pd/p1,8-DAN catalyst electrodes was analyzed using different sweep rates from 0.025 to 0.3 V s^−1^ (Fig. 7A–D). The current densities of the forward peak (*J*_pf_) increased linearly with rising scan rate (*ν*) (figure not shown), while *E*_pf_ and *E*_pb_ shift to a positive potential with a linear relationship between *E*_pf_ and log(*ν*), as shown in Fig. 7 (inset),^27^ which suggests that the electrooxidation of EtOH is an irreversible process.

For each catalyst, the diffusion coefficient (*D*) was calculated according to the following Randles–Sevcik equation.^50,51^*I*_p_ = 2.69 × 10^5^*n*^3/2^*AD*^1/2^*ν*^1/2^*C*where *I*_p_ is the peak current in ampere, *n* is the number of electrons transferred in the rate-determining step, *A* is the electrode surface area, *ν* is the scan rate in V s^−1^, *C* is the solution concentration in mole cm^−3^ and *D* is the diffusion coefficient in cm^2^ s^−1^, which is found by plotting the relation between the square root of the scan rate and the current density. Diffusion coefficients for Pd/p1,8-DAN, Pt/p1,8-DAN, Pd/Pt/p1,8-DAN and Pt/Pd/p1,8-DAN catalysts were found to be 1.29 × 10^−7^, 6.01 × 10^−8^, 4.33 × 10^−7^ and 4.1 × 10^−6^, respectively. This reveals that the charge-transport rate at the Pt/Pd/p1,8-DAN catalyst within the electrode–electrolyte interface is faster than for the other catalysts.

#### 3.3.4. Effect of upper potential limits

Keeping in mind the end goal of assessing the relation between EOR and metal oxide species formed at Pd/p1,8-DAN, Pt/p1,8-DAN, Pd/Pt/p1,8-DAN and Pt/Pd/p1,8-DAN catalysts, the impact of various upper anodic potential limits was explored (figure not shown). In the forward sweep, *J*_pf_ and *E*_pf_ remain unaffected, while in the backward scan, values of *J*_pb_, *E*_pb_, reduction peak current densities (*J*_pr_) and reduction peak potentials (*E*_pr_) were changed. This could be explained by the fact that at lower potential, metal oxides had not been significantly created and so their impact on EOR in the backward scan was minor. Increasing the positive potential limits accelerates metal oxide formation leading to an increase in *J*_pr_. On the other hand, *E*_pb_ had a continuous positive shift and *J*_pb_ improved.^27^

### 3.4. Chronoamperometry

The impact of poisoning the Pt and Pd NPs surfaces was analyzed using the chronoamperometry (CA) technique. A chronoamperometric study was carried out to better understand the electrocatalytic performance and stability of Pd/p1,8-DAN, Pt/p1,8-DAN, Pd/Pt/p1,8-DAN and Pt/Pd/p1,8-DAN catalysts towards EOR (figure not shown) where the potential was held at −0.02 V. By applying potential to each electrode, a steady decrease in current was observed within the initial couple of minutes for all catalysts, followed by the establishment of an almost steady current at longer times. The decrease in current with time could be ascribed to the intermediate poisoning species accumulated during the development of oxidation. The catalytic stability was found to follow the order Pt/Pd/p1,8-DAN > Pd/Pt/p1,8-DAN > Pt/p1,8-DAN > Pd/p1,8-DAN. The results suggested that the Pt/Pd/p1,8-DAN catalyst electrode shows higher catalytic activity and stability towards EOR, proving a superior tolerance to the carbonaceous intermediates generated during the oxidation process, as represented in Table 1.^40,52,53^

## 4. Conclusion

In this work, we have presented a support of a modified p1,8-DAN/GC electrode with Pt, Pd, Pt/Pd and Pd/Pt NPs for EOR. The performances of the resulting catalyst electrodes were considerably enhanced in terms of increasing the catalytic current and depressing the onset potential of EtOH oxidation. DEMS, CV and CA techniques were used to characterize the electrochemical behaviors of the prepared catalysts. Moreover, CA analysis was useful to prove the electrocatalytic performance, catalytic activity and stability of the prepared catalysts toward EOR. Experimental results revealed that the Pt/Pd/p1,8-DAN catalyst demonstrated considerably improved electrocatalytic activity and tolerance to CO poisoning (*J*_Pf_/*J*_Pb_ = 2.6). Additionally, it revealed a superior electrochemically available surface area and faster charge transfer rate at the electrode/electrolyte interface than the other catalysts, making it a smart anode for the manufacture of an EtOH fuel cell.

## Conflicts of interest

There are no conflicts to declare.

## Supplementary Material
